# Preliminary Application of Bilateral Submandibular Horizontal Incision in the Treatment of Upper Cervical Tumors

**DOI:** 10.1111/os.14247

**Published:** 2024-10-20

**Authors:** Jingtao Ji, Guang Dong Chen, Jun Miao

**Affiliations:** ^1^ Department of Spine Surgery of Tianjin Hospital Tianjin China; ^2^ Academy of Medical Engineering and Translational Medicine Tianjin University Tianjin China; ^3^ Department of Orthopaedics Cangzhou Central Hospital Cangzhou Hebei Province China

**Keywords:** bilateral submandibular horizontal incision, reconstruction, tumor, upper cervical spine

## Abstract

The upper cervical spine has a complex anatomical structure, making anterior surgical approaches challenging and prone to complications. This study aims to explore the use of bilateral submandibular incisions to provide safer and more convenient exposure of the upper cervical spine and to assess the feasibility of this approach for anterior surgical treatment of complex upper cervical diseases. From November 2019 to August 2021, three patients with malignant tumors of the upper cervical spine were subjected to an anterior–posterior combined approach for cervical tumor resection. The cohort consisted of one male and two females, aged between 41 and 51 years. The anterior approach began with a submandibular incision, followed by blunt dissection through the prevertebral muscles to expose the diseased vertebra. Subsequently, the diseased vertebra was excised, and either a titanium cage or a pre‐customized 3D‐printed artificial vertebral body was implanted anteriorly. Then, posterior fixation of the cervical spine was performed using pedicle screws to provide additional stability. Follow‐up ranged from 8 to 34 months. All patients experienced varying degrees of pain relief within 24 hours post‐operation. Frankel grading showed improvement by at least one grade in all three cases. Regular X‐ray and magnetic resonance imaging examinations revealed no tumor recurrence or involvement of adjacent vertebrae in the surgical area. The anterior bilateral submandibular horizontal incision approach offers comprehensive exposure of the anatomical structures of the upper cervical spine. This approach introduces a new option for the anterior treatment of upper cervical spine diseases.

## Graphical abstract

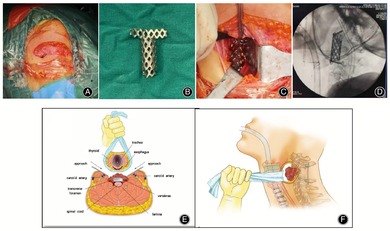

## Introduction

The upper cervical spine typically refers to the atlas (C1), axis (C2), and associated structures such as the articular processes, articular capsules, and ligaments. The anatomical structures in this region are intricate, adjacent to vital organs, nerves, and blood vessels, and are hindered by the mandible. Consequently, surgery involving anterior exposure presents challenges, owing to increased intraoperative bleeding and a higher rate of postoperative complications; thus, the anterior region has historically been regarded as a surgically restricted zone. For fractures and dislocations of the atlas and axis, posterior decompression and fusion with pedicle screw fixation are commonly employed. However, in cases of severe deformities, tumors, infections, and other conditions causing anterior compression of the spinal cord, anterior decompression and fixation are necessary. In atlantoaxial surgeries, the transoral pharyngeal route is frequently utilized for anterior access.

By making incisions through the mandible and the tongue, surgeons can achieve adequate exposure from the clivus to C5 of the skull base.^1^ This approach facilitates odontoid process release or the resection of tumors, tuberculosis, and bony compressions in this region, enabling direct anterior decompression. However, this surgical procedure is associated with a high postoperative infection rate.^2^ Within the first 2 weeks of surgery, patients may require nasogastric tube feeding, and complications such as palatopharyngeal dysfunction, temporomandibular joint impairment, and difficulties in swallowing and speaking may occur.^3^

Furthermore, the retropharyngeal approach is also a commonly used method, entailing an incision that extends from below the right angle of the jaw to the lower margin of the mandible, which is aligned parallel to the horizontal plane of the mandible. This approach allows exposure from the occipital bone slope to C3.^4^ Owing to the absence of oral cavity manipulation, the postoperative infection rate is lower, reducing some complications associated with oral approaches. However, this approach involves complex anatomical structures, including the submandibular gland, digastric muscle, sublingual nerve, branches of the facial nerve, lingual artery, superior laryngeal artery, superior laryngeal nerve, recurrent laryngeal nerve, superior thyroid vessels, and vertebral artery. Damage to these important structures can result in corresponding complications, requiring the surgeon to possess extensive experience.^5^, ^6^ In operations involving contralateral structures, excessive traction on the esophagus and trachea is necessary, which may lead to esophageal functional impairment, limiting the widespread application of this approach.

Since 2019, our hospital has attempted a novel approach for exposing the upper cervical spine in patients with C2 and C3 vertebral tumors. We utilized a bilateral submandibular horizontal incision, which is a modification of the traditional Smith‐Robinson approach by elevating the incisions closer to the mandible's lower border. The standard Smith‐Robinson incision typically provides exposure only up to the C3 vertebra. However, by elevating the bilateral incisions, we could successfully resect and reconstruct tumors in the upper cervical spine. This procedure, combined with posterior pedicle screw fixation, achieves the goal of restoring spinal stability. This approach has several advantages: (I) It does not require mandibular splitting or exposure of important neurovascular structures, thereby reducing surgical complications. (II) The bilateral approach provides sufficient exposure of the anterior structures of the upper cervical spine, reducing excessive traction on the trachea and esophagus. (III) The bilateral approach offers a larger operating space, facilitating the implantation of T‐shaped titanium cages or 3D‐printed artificial vertebrae after the resection of the diseased vertebrae, with clearer visualization. This method offers a new option for the anterior treatment of upper cervical spine diseases.

## Materials and Methods

### General Clinical Data

Inclusion Criteria: (i) Malignant spinal tumors confirmed by imaging or pathology. (ii) Imaging shows intradural space‐occupying lesions, and patients have symptoms of nerve compression, which may be accompanied by or without pain and pathological vertebral fractures. (iii) Patients with primary malignant spinal tumors and metastatic malignant tumors (preoperative Tomita score of 2–5, or modified Tokuhashi score of 9–15).

Exclusion Criteria: (i) Patients diagnosed by imaging or confirmed by pathology but without symptoms of nerve compression; (ii) patients with metastatic tumors with a preoperative Tomita score of 8–10 or a modified Tokuhashi score of 0–7 who cannot tolerate surgery; (iii) patients with metastatic tumors with a preoperative Tomita score of 6–7 or a modified Tokuhashi score of 9–11, with multiple metastases requiring palliative surgery; (iv) patients with uncontrolled acute or active infections in any part of the body.

From November 2019 to August 2021, a combined anterior–posterior approach was employed for cervical tumor resection in three patients with malignant tumors of the upper cervical spine. The anterior approach involved a bilateral submandibular horizontal incision to expose the upper cervical spine. In one case, a 3D‐printed artificial vertebral body was used for anterior column reconstruction, while in two cases, reconstruction was performed using titanium mesh and allograft bone. Posterior treatment involved pedicle screw fixation. The cohort consisted of one male and two females, aged between 41 and 51 years, with an average age of 47.3 years. The affected vertebrae were the axis (C2) in two cases and the body of the third cervical vertebra (C3) in one case. Primary tumors were seen in one case of chordoma and in one case of plasmacytoma; the third case involved lung cancer with bone metastasis (Tomita score preoperatively: 5, Tokuhashi score: 10) (Tianjin Hospital Medical Ethics Committee: 2024YLS054).

All three patients in this group experienced preoperative neck pain, predominantly at night. The preoperative visual analog scale (VAS) scores ranged from 5 to 8, with an average of 6.7. Each patient presented with varying degrees of symptoms related to spinal cord or nerve root compression. In the case involving a C3 spinal cord tumor, the patient exhibited bilateral upper limb muscle weakness (grade 4) and unsteady walking and was classified as Frankel Grade D. In two cases involving C2 vertebral body tumors, the patients reported bilateral numbness in the hands, with normal muscle strength and sensation in the limbs, classified as Frankel Grade E. The affected vertebrae in all three cases exhibited lytic bone destruction, with preoperative spinal instability neoplastic scores ranging from 10 to 12, averaging 10.7.

All three patients underwent preoperative x‐ray, computed tomography (CT), and magnetic resonance imaging (MRI) examinations, with two of them also undergoing positron emission tomography‐CT scans. Preoperative x‐rays revealed significant lytic bone destruction of the vertebral bodies with associated pathological vertebral fractures. CT scans showed lytic bone destruction in the affected vertebrae, with one patient exhibiting soft tissue tumor occupancy within the spinal canal and another showing the presence of soft tissue tumors around the vertebral body. On MRI T1‐weighted images, the areas of bone destruction appeared as low‐signal tumor tissue, whereas on T2‐weighted images, they exhibited high signal intensity with a mixed fat signal. In one case, the soft tissue mass appeared as a mixed signal with distinct borders, occupying the spinal canal and causing compression of the spinal cord.

### Surgical Technique

#### Preoperative Preparation and Anesthesia

All patients were subjected to preoperative localization of the affected segment through C‐arm fluoroscopy. The incision was a curved cut, parallel to the lower jaw and approximately 1 cm below the mandible. This incision extended bilaterally to the inner edge of the sternocleidomastoid muscle, as marked on the skin surface. General anesthesia was administered to all three patients.

#### Surgical Exposure

The patient was positioned supine after routine disinfection and draping. Following surface markings, the skin, subcutaneous tissues, and deep fascia were incised. The incision extended through the platysma muscle, beneath the digastric muscle, and blunt dissection proceeded along the medial aspect of the carotid sheath, adjacent to the inner edge of the sternocleidomastoid muscle, allowing access to the anterior neck region. The prevertebral fascia was carefully dissected, and bilateral gauze pads were alternately used to gently separate the anterior neck tissues toward the head, exposing the odontoid process slope and the anterior arch of the atlas. Localization needles were inserted into the intervertebral spaces, and their positions were confirmed *via* C‐arm fluoroscopy.

##### Affected Vertebrae Resection

The anterior fibrous ring above and below the affected vertebrae was incised using a sharp knife. A curette was used to remove the intervertebral disc and cartilaginous endplate. Subsequently, bone rongeurs and Kerrison punches were employed to excise the diseased vertebra and the posterior longitudinal ligament. In cases involving the odontoid process, it was necessary to remove it to expose the dura mater while preserving the transverse processes on both sides to avoid injuring the vertebral artery.

#### Vertebral Body Reconstruction

**FIGURE 1:**
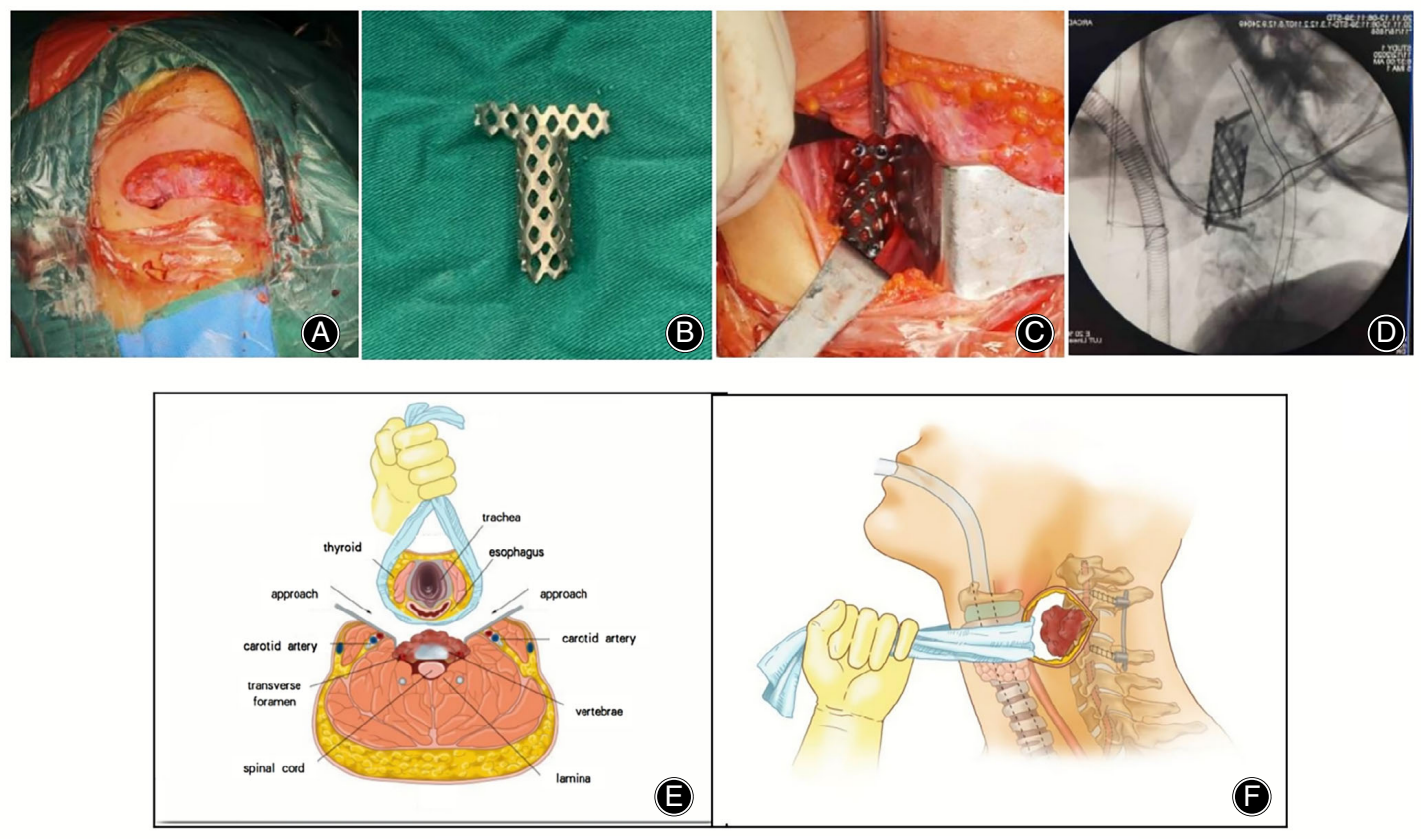
The anterior cervical incision. (A) Surgical incision. (B) Titanium cage cut extracorporeally. (C) Implantation of the pre‐cut titanium cage into the anterior part after implanting allogeneic bone. (D) Fluoroscopic position of the C‐shaped arm. (F,G) Surgical illustration.

The upper and lower cartilaginous endplates of the diseased vertebra were removed using a curette while preserving the bony endplates. Subsequently, an appropriately sized titanium cage or a pre‐customized 3D‐printed artificial vertebral body was implanted into the anterior neck region after grafting with allograft bone. Following satisfactory C‐arm fluoroscopy and internal fixation, a drainage tube was kept in place, and the wound was closed in layers (Figure 1).

#### Posterior Fixation

**FIGURE 2:**
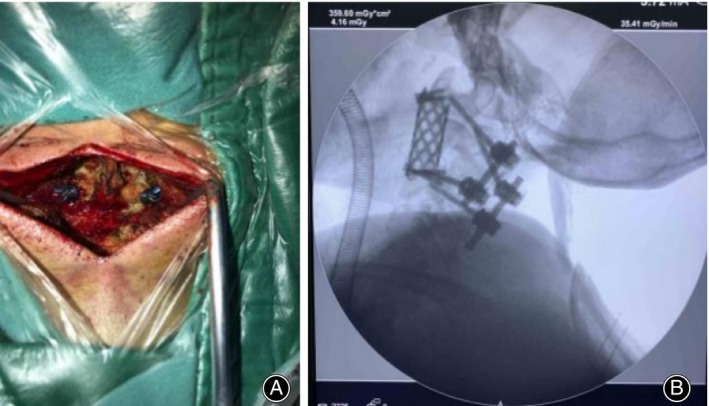
Posterior cervical incision. (A) Insertion of pedicle screws in the posterior aspect of C1 and C2 vertebrae. (B) Fluoroscopic position of the C‐shaped arm.

The patient was turned into a prone position after routine disinfection and draping. A midline incision was made along the spinous processes of the cervical vertebrae (Figure 2). The incision passed through the skin, subcutaneous tissues, and deep fascia. Bilateral subperiosteal dissection was conducted to expose the paraspinal muscles up to the outer edge of the transverse processes. Entry points for pedicle screws were selected at the midpoint of the upper outer quadrant on each side. Pedicle screws were inserted into the vertebral arch using a pedicle probe, following confirmation of the bony pathway with an awl. Following satisfactory C‐arm fluoroscopy and internal fixation, titanium rods and caps were connected. The pedicle screws were tightened from the posterior aspect, and one drainage tube was retained. The wound was closed in layers.

#### Postoperative Management

In all patients, wound drainage was less than 50 mL, leading to the removal of the drainage tube. External cervical immobilization with a neck brace was necessary before mobilization, and it was accompanied by the administration of neurotrophic and analgesic medications for treatment.

##### Observational Indicators

Patient pain improvement was assessed through a comparison of preoperative and postoperative (24 h and 3 months) VAS scores. Neurological function improvement was evaluated using the Frankel grading system.

All three patients in this group underwent postoperative x‐ray and CT examinations. Follow‐up x‐ray evaluations were conducted at outpatient clinics to assess the stability of internal fixation, while MRI was employed to monitor for local tumor recurrence.

## Results

### General Results

Blood loss ranged from 500 to 1200 mL, averaging 833.3 mL. The drainage tube was typically removed within 3–5 days post‐operation, and the drainage volumes ranged from 380 to 850 mL. The average drainage was 665.0 mL.

### Pain Relief

Within 24 h post‐operation, all three patients experienced varying degrees of pain relief. The preoperative VAS scores for the patients ranged from 5 to 8, averaging 6.7. After 24 h, the VAS scores ranged from 2 to 4, averaging 2.7. Three months post‐operation, the VAS scores ranged from 1 to 3, with an average of 1.7.

#### Neurological Function Recovery

In one case, a patient with a C3 spinal cord tumor was initially classified as Frankel Grade D before surgery, but by the last follow‐up, their classification had improved to Grade E. The other two patients were initially classified as Frankel Grade E, and there was no change in their Frankel grading at the last follow‐up.

### Oncological Results

The cases in this group were followed up for 8 to 34 months, with an average of 18.7 months. One patient with plasma cell myeloma received postoperative radiotherapy with a dose of 40 Gy 2 weeks after surgery. Another patient, diagnosed with lung cancer, underwent targeted drug therapy following surgery. All three patients received bisphosphonate treatment postoperatively. At the last follow‐up, two patients were alive, while one patient with lung cancer passed away owing to brain metastasis 8 months after operation. The remaining two patients did not show metastasis to other sites during the follow‐up period. During follow‐up, there was no recurrence at the surgical site in any patient.

### Radiological Results

#### Local Tumor Control

The cases in this group were followed up for 8 to 34 months, with regular x‐ray and MRI examinations conducted every 3 months. No signs of tumor recurrence at the surgical site or involvement of adjacent vertebrae were observed in any of the cases during follow‐up.

#### Internal Fixation Status

After anterior vertebral body resection surgery in the three patients: In two cases of vertebral body tumors, titanium mesh was used for reconstruction with allograft bone. In one patient, the titanium mesh was fixed with four screws, while in another patient, the titanium mesh was not secured with screws.

In one case of a C3 vertebral body tumor, reconstruction was achieved using a 3D‐printed artificial vertebral body (Beijing Aikangyicheng Medical Equipment Co., Ltd., China).

In two cases of vertebral body tumors, posterior fixation was performed using four pedicle screws secured to the vertebral arch and C3 vertebral body. In another case of a C3 vertebral body tumor, posterior fixation was performed using five pedicle screws fixed to the vertebral arch, C3, and C4 vertebral bodies.

Postoperative x‐rays and CT scans for all patients revealed satisfactory positioning of the anterior titanium mesh or 3D‐printed artificial vertebral body and the pedicle screws. Throughout the follow‐up period, there was no evidence of implant subsidence or loosening of the pedicle screws in any patient.

### Pathological Results

All three patients were subjected to pathological examinations, and the results revealed two cases of primary malignant tumors: one chordoma and one plasma cell myeloma. The third case involved bone metastasis from lung cancer.

### Complications

**FIGURE 3:**
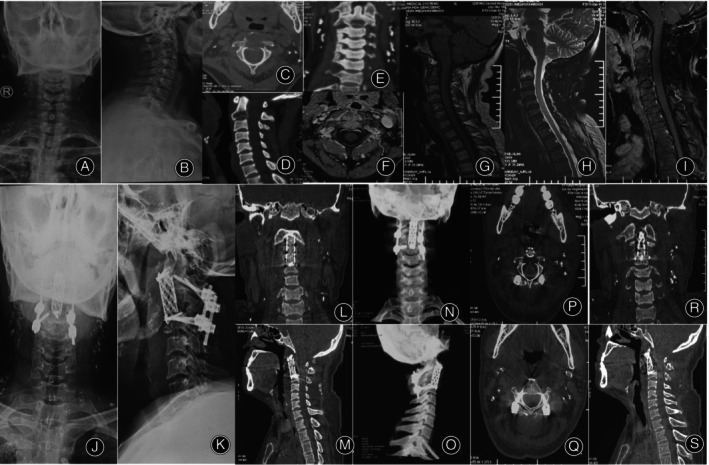
Female, 50 years old, with lung cancer metastasis to the bones. (A,B) Preoperative x‐rays showing lesions in the C2 vertebra. (C–E) Preoperative CT scans revealing bone destruction in the C2 vertebra, posterior vertebral bone defect, and spinal cord compression. (F–I) Preoperative lumbar spine MRI demonstrating C2 vertebral bone destruction, formation of soft tissue tumor around, and compression of the dural sac. (J,K) The x‐ray taken 1 week postoperatively showed that the cervical pedicle screws and the titanium cage were stable. (L–O) The CT scan 1 week postoperatively showed adequate spinal canal decompression, with the titanium cage and pedicle screws remaining stable. (P–S) The CT scan 6 months postoperatively showed no significant tumor recurrence in the surgical area, and the titanium cage and pedicle screws remained stable. CT, computed tomography; MRI, magnetic resonance imaging.

**FIGURE 4:**
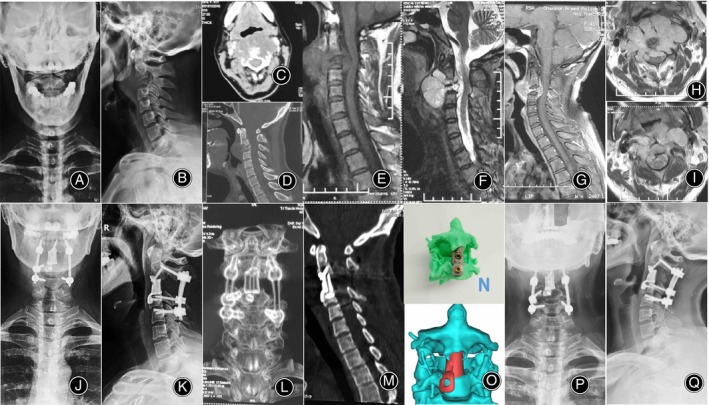
Female, 41 years old, with chordoma. (A,B) Preoperative x‐rays showing lesions in the C3 vertebra. (C,D) Preoperative CT scans revealing bone destruction in the C3 vertebra, and spinal cord compression. (E–I) Preoperative lumbar spine MRI demonstrating C3 vertebral bone destruction, formation of soft tissue tumor around, and compression of the dural sac. (J,K) The x‐ray taken 1 week postoperatively showed that the cervical pedicle screws and the 3D printed artificial vertebral body were stable. (L,M) The CT scan 1 week postoperatively showed adequate spinal canal decompression, with the 3D printed artificial vertebral body and pedicle screws remaining stable. (N,O) 3D printed model and 3D software reconstructed model. (P,Q) The x‐ray 2 years after surgery showed no significant tumor recurrence in the surgical area, and the 3D printed artificial vertebral body and pedicle screws remained stable. CT, computed tomography; MRI, magnetic resonance imaging.

**FIGURE 5:**
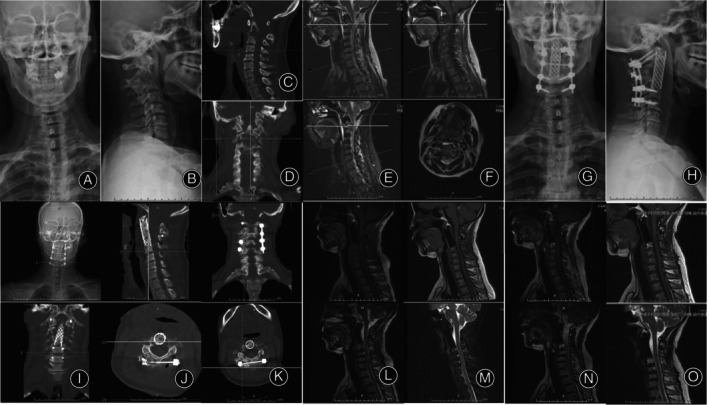
Male, 51 years old, with plasmacytoma. (A,B) Preoperative x‐rays showing lesions in the C2 vertebra. (C,D) Preoperative CT scans revealing bone destruction in the C2 vertebra. (E,F) Preoperative lumbar spine MRI demonstrating C2 vertebral bone destruction. (G,H) The x‐ray taken 1 week postoperatively showed that the cervical pedicle screws and the titanium cage were stable. (I,J) The CT scan 1 week postoperatively showed adequate spinal canal decompression, with the titanium cage and pedicle screws remaining stable. (K) The CT scan taken 6 months postoperatively revealed adequate decompression of the spinal canal, with the titanium cage and pedicle screws remaining stable. (L) The MRI taken 6 months postoperatively showed no significant tumor recurrence in the surgical area, with the titanium cage and pedicle screws remaining stable. (M) The MRI taken 1 year postoperatively showed no significant tumor recurrence in the surgical area, with the titanium cage and pedicle screws remaining stable. (N) The MRI taken 2 years postoperatively showed no significant tumor recurrence in the surgical area, with the titanium cage and pedicle screws remaining stable. (O) The MRI taken 34 months postoperatively showed no significant tumor recurrence in the surgical area, with the titanium cage and pedicle screws remaining stable. CT, computed tomography; MRI, magnetic resonance imaging.

There were no occurrences of severe complications such as nerve injury, infection, or loosening of internal fixation in the three patients post‐operation (Figures 3, 4, 5).

## Discussion

The bilateral submandibular horizontal incision offers a straightforward and convenient approach for treating upper cervical spine diseases. This method provides complete exposure of the anatomical structures of the upper cervical spine without excessive traction on the trachea and esophagus, thus preventing damage to critical anatomical structures. Moreover, this technique provides sufficient space for the reconstruction of the upper cervical spine. It represents an innovative anterior surgical approach for treating upper cervical spine diseases.

### Advantages of the Bilateral Submandibular Horizontal Incision Approach in Exposing Diseased Vertebrae

The bilateral submandibular incision entails accessing the anterior neck through the space beneath the digastric muscles, the region along the inner side of the sternocleidomastoid muscle, and the sheath of the carotid artery. In some patients, this approach provides direct exposure to the C2/3 intervertebral disc, allowing immediate visibility of the vertebral body of the axis (C2) and the anterior arch of the atlas (C1) upon the displacement of the anterior cervical fascia toward the head. However, in some patients, exposure may be restricted to the C3 vertebra, requiring a more pronounced head position to reduce obstruction from the mandible. Despite this, clear visualization of the vertebral body of the axis and the anterior arch of the atlas can still be achieved through the displacement of the anterior cervical fascia after the extension of the head.

This approach eliminates the need for an intraoral route, thus avoiding the requirement to split the mandible and incise the posterior pharyngeal wall, which reduces surgical complexity and infection risk. Furthermore, despite the transverse nature of the skin incision beneath the mandible, it is sufficiently long to fully expose the inner edge of the sternocleidomastoid muscle. Subsequently, the conventional anterior neck approach (Smith–Robinson approach) is employed, with alternating use of gauze to separate the anterior neck tissues toward the head until the vertebral body of the axis (C2) and the anterior arch of the atlas (C1) are exposed. There is no need to expose vital structures such as the submandibular gland, sublingual nerve, facial nerve, lingual artery, superior laryngeal artery, superior laryngeal nerve, and recurrent laryngeal nerve. This simplifies the surgical approach and reduces the risk of damage to these critical structures.

Given its bilateral nature, this approach offers superior exposure to the anterior aspect of the upper cervical spine. Particularly advantageous for complex conditions such as tumors or tuberculosis, the approach enables more thorough visualization of soft tissue masses, thereby reducing the risk of contaminating surrounding normal tissues. Additionally, the bilateral approach reduces excessive traction on the trachea and esophagus. This is particularly advantageous for the thorough and safe release of adhesions in the anterior neck region, thereby lowering the risk of injury to normal structures such as the trachea and esophagus.

In conditions such as tumors and tuberculosis, the bilateral approach is more advantageous during vertebral resection. This is particularly evident in the handling of transverse processes and protection of the vertebral artery, where clearer exposure helps prevent damage. Regarding structures behind the affected vertebra, particularly in cases where lesions adhere to the dura mater, the bilateral approach provides enhanced visualization of posterior structures. This facilitates safer separation from various angles, reducing the risk of dural damage from visual blind spots and preventing cerebrospinal fluid leakage.

In this group of patients, the average surgery duration was 260.0 min, with an average blood loss of 833.3 mL. Compared to the transoral approach, this technique resulted in less trauma, reduced blood loss, and shorter surgery times. None of the three patients experienced complications related to upper cervical neurovascular injury postoperatively. Additionally, this approach offers a shorter learning curve, simpler operation, and a lower risk of significant neurovascular damage compared to the transoral approach, making it more favorable for wider adoption.

### Advantages of the Bilateral Submandibular Horizontal Incision Approach in Vertebral Reconstruction

After the removal of the diseased vertebra, fixation with screws on the posterior pedicle alone may not provide sufficient strength, necessitating anterior internal fixation and reconstruction.^7^, ^8^ However, owing to the complex anatomical structures of the upper cervical spine and its proximity to vital neurovascular structures, unilateral approaches have limited incision scope. Implantation and fixation from the anterior aspect of the cervical spine are challenging, especially given the narrow space anterior to the C1 vertebra. For reconstructions involving a T‐shaped titanium cage or a custom 3D‐printed artificial vertebra, adequate exposure of the anterior aspect of the C1 vertebra is crucial to ensure sufficient contact surface for cranial fixation of the implant.

Compared with titanium cages, 3D‐printed artificial vertebrae offer advantages in terms of both subsidence rate and fusion rate,^9^, ^10^, ^11^, ^12^, ^13^, ^14^, ^15^, ^16^ contributing to their increasing popularity in upper cervical spine reconstruction. However, 3D‐printed artificial vertebrae have larger volumes, making it exceedingly challenging to adjust their position after unilateral insertion. This difficulty is particularly evident during the placement of contralateral screws and the protection of contralateral arteries, which often require more time and increase the risk of damage to arteries, nerves, and even the trachea and esophagus.

Bilateral approaches facilitate symmetrical exposure through anterior dissection, offering a larger operating space by fully mobilizing the anterior cervical structures. Specifically, the exposure of both sides anterior to the C1 vertebra is more comprehensive. During the implantation of T‐shaped titanium cages or 3D‐printed artificial vertebrae, there is reduced interference with surrounding nerves, blood vessels, trachea, and esophagus. With ample space for symmetrical fixation on both sides at the cranial end of the implant, visibility is clear, and adjusting screw direction during insertion is straightforward, reducing repetitive operations and ensuring screw stability. Furthermore, adjusting the position of the implant after bilateral insertion is more convenient with bilateral approaches. This allows for full exposure and protection of surrounding critical anatomical structures, and the ideal fixation position is achieved. This approach can shorten surgical time and reduce blood loss.

The anterior bilateral submandibular incision is suitable for exposing tumors, infectious diseases, or severe atlantoaxial deformities involving C1–C3. This approach is straightforward and convenient, avoiding the exposure of critical anatomical structures, thereby reducing the risk of injury. The bilateral approach provides comprehensive exposure of the anterior structures of the upper cervical spine, facilitating lesion resection. Additionally, the approach offers a larger operating space, simplifying the implantation of T‐shaped titanium cages or 3D‐printed artificial vertebrae after the resection of the diseased vertebrae, with enhanced visualization. This represents a novel anterior surgical approach for treating upper cervical spine diseases.

In this group of patients, one case of metastatic cancer and one case of plasmacytoma underwent vertebral reconstruction with a titanium cage following the tumor resection. Another patient with a chordoma, due to the long survival period and the high likelihood of local recurrence after surgery, underwent a more extensive tumor resection. To achieve better stability, higher fusion rates, and lower subsidence rates, this patient opted for anterior reconstruction using a 3D‐printed artificial vertebral body.

### Surgical Technique and Considerations for a Double Submandibular Incision Approach

1. Selection of Incision Location: The bilateral submandibular horizontal incision is a symmetrical horizontal incision parallel to the mandible, positioned 1 cm below the mandibular border. If the incision is made too high, it can result in damage to critical structures such as the submandibular gland, facial nerve, superior laryngeal nerve, and recurrent laryngeal nerve during the separation of anterior cervical soft tissues. This can lead to postoperative sensory abnormalities and functional limitations in the innervated areas, as well as significant intraoperative bleeding due to injury to vessels like the lingual artery, superior laryngeal artery, and superior thyroid vessels. Conversely, if the incision is too low, the skin and soft tissues of the head may obstruct the view, making it difficult to expose the C2 vertebra or limiting the surgical field, thereby complicating tumor resection and anterior prosthetic implantation.
2. Protection of the Trachea and Esophagus: The bilateral submandibular horizontal incision is a modified approach of the Smith‐Robinson technique. Thus, special care is required to protect the trachea and esophagus during the procedure. During unilateral operations, a retractor should gently pull the trachea and esophagus toward the opposite side. Once both sides are fully exposed, gauze is threaded between the esophagus and vertebral body to lift and protect the trachea and esophagus, providing adequate space for vertebral resection and reconstruction.
3. Protection of the Vertebral Artery: The vertebral artery runs through the transverse foramen on either side of the vertebral body, making exposure and protection challenging, especially in the upper cervical spine, where anatomical variations of the vertebral artery are common. Intraoperative injury to this artery can be fatal. The bilateral submandibular horizontal incision approach offers better exposure, reducing the risk of vertebral artery injury when performing en bloc vertebral resection. However, if whole vertebral body resection is required, it is recommended to first expose, protect, and mark the vertebral artery through a posterior approach to facilitate the anterior procedure. When the vertebral artery is encased by the tumor, a posterior approach should first ligate and sever the normal segments of the vertebral artery above and below the tumor on the affected side to facilitate anterior tumor resection.

### Limitations and Strengths

In this group of three patients, one case involved a metastatic tumor, one case was a plasmacytoma, and one had a chordoma causing severe vertebral destruction. Consequently, all three patients underwent piecemeal resection of the anterior tumors. In future studies, this approach may be more suitable for the en bloc resection of primary malignant spinal tumors. Additionally, owing to the low incidence of upper cervical tumors or tuberculosis, the number of cases in this group was relatively small. However, this approach provides a more convenient and safer method for exposing upper cervical lesions. More cases are needed to further investigate the long‐term efficacy of the bilateral technique, and continuous improvement and refinement in future research are necessary.

## Conclusion

The anterior bilateral submandibular horizontal incision approach offers comprehensive exposure of the anatomical structures of the upper cervical spine without excessive traction on the trachea and esophagus, thereby minimizing the risk of damage. Bilateral exposure also enhances visualization of the vertebral artery, aiding in its protection. This approach offers a new option for the anterior treatment of upper cervical spine diseases.

## Conflict of Interest

The authors declare no conflicts of interest.

## Ethics Statement

All clinical investigations had been conducted according to the principles expressed in the Declaration of Helsinki. This study was conducted with approval from the Ethics Committee of Tianjin Hospital. Informed consent to participate in the study was obtained from the participant.

## Author Contributions

Jun Miao conceived the original ideas of this manuscript. Jingtao Ji wrote the manuscript. Jingtao Ji, Jun Miao, and Guangdong Chen jointly performed the surgical operation. Jingtao Ji and Guangdong Chen were responsible for image production. All authors read and approved the final manuscript.
